# *Clostridioides difficile* positivity rate and PCR ribotype distribution on retail potatoes in 12 European countries, January to June 2018

**DOI:** 10.2807/1560-7917.ES.2022.27.15.2100417

**Published:** 2022-04-14

**Authors:** Valerija Tkalec, Virginie Viprey, Georgina Davis, Sandra Janezic, Béatrice Sente, Nathalie Devos, Mark Wilcox, Kerrie Davies, Maja Rupnik, on behalf of the COMBACTE-CDI consortium

**Affiliations:** 1 National Laboratory for Health, Environment and Food, Maribor, Slovenia; 2 Faculty of Medicine, University of Maribor, Maribor, Slovenia; 3 Leeds Institute of Medical Research, University of Leeds, United Kingdom; 4 GSK Vaccines, Rixensart, Belgium; 5 European Study Group *Clostridioides difficile*, ESCMID; 6 The members of the COMBACTE-CDI consortium are acknowledged at the end of the article

**Keywords:** *Clostridium difficile*, *Clostridioides difficile*, food chain, food, vegetables, potato, One Health

## Abstract

**Background:**

While human-to-human transmission of *Clostridioides difficile* occurs often, other infection sources, including food, animals and environment, are under investigation.

**Aim:**

We present a large study on *C. difficile* in a food item in Europe, encompassing 12 European countries (Austria, France, Greece, Ireland, Italy, the Netherlands, Poland, Slovakia, Spain, Sweden, Romania and the United Kingdom).

**Methods:**

Potato was selected because of availability, ease of sampling and high *C. difficile* positivity rates. Identical protocols for sampling and isolation were used, enabling a direct comparison of the *C. difficile* positivity rate.

**Results:**

From *C. difficile*-positive potato samples (33/147; 22.4%), we obtained 504 isolates, grouped into 38 PCR ribotypes. Positivity rates per country varied (0–100%) and were at least 10% in 9/12 countries. No geographical clustering of samples with high positivity rates or in PCR ribotype distribution was observed. The most frequently detected PCR ribotypes (014/020, 078/126, 010 and 023) are also commonly reported in Europe among human clinically relevant isolates, in animal isolates and in the environment. Whole genome sequencing revealed several genetically related strain pairs (Spain/RT126, France/RT010, Austria and Sweden/RT276) and a cluster of very similar strains in RT078/126.

**Conclusion:**

Our results suggest, the high potato contamination rates could have public health relevance. They indicate potatoes can serve as a vector for introducing *C. difficile* spores in the household environment, where the bacterium can then multiply in sensitive hosts with disrupted or unmature microbiota. Potato contamination with PCR ribotypes shared between humans, animals and soil is supportive of this view.

## Introduction

*Clostridioides difficile* infection (CDI) is a notable cause of infectious diarrhoea worldwide. In Europe, the estimated number of CDI cases in 2011–12 was 123,997 (95% confidence interval (CI): 61,018–284,857), based on a survey of healthcare-associated infections performed by the European Centre for Disease Prevention and Control (ECDC) [1]. In 2016, as part of *C. difficile* surveillance performed by ECDC, 556 hospitals from 20 countries covering 24 million patient-days reported 7,711 CDI cases [2]. The symptoms can range from mild diarrhoea to potentially fatal pseudomembranous colitis. While historically regarded as a typical healthcare infection, community CDI is increasingly recognised [3].

In CDI, human-to-human transmission plays a major role, but other infection sources and transmission routes are under investigation. *C. difficile* has been repeatedly isolated from various foods worldwide, and it is feasible that some foods could be important vectors for its widespread dissemination [3]. Some important healthcare-associated *C. difficile* PCR ribotypes (RT) such as RT 027 and RT 001/072 tend to spread clonally within a single hospital, region or country, while others such as RT 014, RT 002 and RT 015 do not exhibit country-based clustering and are most likely disseminated across Europe by other sources possibly including the food chain [4]. Confirmed cases of food-associated CDI have so far not been described [3].

Existing evidence suggests that potatoes, which represent a major staple food consumed worldwide, could contribute to the spread of *C. difficile*. Potatoes have the highest *C. difficile* contamination rates among all vegetables tested to date; the proportion of *C. difficile*-positive retail potato samples ranges from 25.7% (18/70) to 53.3% (24/45) [5,6]. By contrast, the highest positivity rate in other types of vegetables such as leaf vegetables, ginger, sprouts and ready-to-eat salads is 9.4% [5-8] and in meats and meat products, reported positivity rates are typically below 20% [8,9]. Additionally, diverse and clinically relevant *C. difficile* PCR ribotypes have been previously recovered from potatoes. Certain PCR ribotypes such as RT 014/020, which are suggested to spread by non-clonal transmission networks [4], are among those often detected on potatoes [5,6]. Furthermore, potatoes are frequently imported and exported between countries. A previous study from Slovenia reported that 78.9% (15/19) of *C. difficile*-positive retail potatoes were imported from more than 10 countries on three different continents [6].

Here we present the results of a European-wide study on *C. difficile* contamination of retail potatoes. Identical protocols for sampling and isolation were used for all 12 studied countries, enabling a direct comparison of the positivity rates of *C. difficile* on potatoes.

## Methods

### Study setting

COMBACTE-CDI (Combatting Bacterial Resistance in Europe-CDI) is a large European consortium of CDI experts from eight academic and research organisations collaborating with six industrial partners (The European Federation of Pharmaceutical Industries and Associations (EFPIA) members). One of the main goals of COMBACTE-CDI is to generate a detailed understanding of the *C. difficile* epidemiology in Europe, which includes providing up-to-date information on *C. difficile* in food across Europe. To achieve this goal COMBACTE-CDI has collected clinical, animal and food samples in 12 European countries. We refer to the study presented here as the ‘COMBACTE-CDI potato study’, to distinguish this work from other investigations of *C. difficile* by the same consortium.

Potatoes were selected as a standardised food of non-animal origin to be sampled because of previously reported high *C. difficile* contamination rates.

Although designed as prospective point prevalence study, the entire sampling interval was 6 months, while individual participants each carried out the sampling within a few days.

### Potato sample collection

Potatoes were sampled from 12 countries distributed into the four European regions defined according to the United Nations Geoscheme [10]: East: Poland, Romania, Slovakia; West: Austria, France, Netherlands; North: the United Kingdom (UK), Ireland, Sweden; South: Greece, Italy, Spain. All samples were collected between January and July 2018 (Supplementary Table S1: Information on sampling and *C. difficile*-positive samples in recruited European countries).

Sampling was organised by NLZOH (National Laboratory for Health, Environment and Food, Maribor, Slovenia). Sampling packages were prepared and distributed to one to six contact persons per country. Altogether, 22 of 27 of these recruited contact persons were experienced microbiologists and were familiar with aseptic swabbing techniques. All sampling was done in urban areas.

The sampling package included complete materials necessary for aseptic swabbing for five potato samples and return shipping. Package contents for sampling included underpads, gloves, sterile Polywipe sponge swabs (Medical Wire and Equipment (MWE), Corsham, UK), sterile plastic blender bags marked with the sample number (BagMixer, Interscience, St Nom la Bretèche, France), plastic food bags, small paper envelopes marked with the sample number, one large padded envelope and one shipping envelope provided by an express delivery service. Sample packaging also included a detailed potato protocol to standardise the process. Collaborators were asked to randomly obtain retail potatoes and to swab three tubers per sample purchase, with a tuber size of 5 to 10 cm by 3 to 5 cm. In addition, participants received questionnaires to provide information on the date and place of sampling, the origin of potatoes if available, and an evaluation of the amount of soil contamination on the sampled potatoes distributed into three categories (visibly clean, or moderately or excessively covered with soil).

Per protocol, each individual sponge swab was placed in a pre-marked sterile blender bag, then in a plastic food bag and lastly in a sample-marked paper envelope to avoid any cross-contamination and mislabelling. All five swabs and a completed questionnaire were then packed in a large, padded envelope and returned to NLZOH.

In addition, some potato samples (n = 30; 13 from Austria, nine from Italy, four from Slovakia, two from Romania, one from France and one from the UK) were collected by members of NLZOH while travelling in those countries. In these cases, at least three potato tubers per sample were purchased, double packed in a plastic bag, brought to the NLZOH laboratory and aseptically swabbed, according to the standard protocol.

### *C. difficile* cultivation, PCR ribotyping and toxinotyping

All collected sponge swabs were cultured according to the protocol by Tkalec et al. [6]. Briefly, swabs were enriched in selective BHIST broth (brain heart infusion broth supplemented with *C. difficile* selective supplement (Oxoid, Basingstoke, UK) with 0.1% L-cysteine, 0.5% yeast extract and 0.1% taurocholic acid); enrichment cultures were treated with ethanol shock and plated on chromogenic plates (CHROM ID CDIFF, bioMérieux, Lyon, France). Presumptive *C. difficile* colonies (black or white) were sub-cultured if the colony morphology was suggestive of *C. difficile*. Identification was confirmed by mass spectrometry (MALDI-TOF Biotyper System; Bruker, Bremen, Germany).

Up to 12 isolates per *C. difficile*-positive sample were analysed by screening with crude PCR ribotyping in order to identify identical PCR ribotypes. Screening was performed using a modified PCR ribotyping protocol by Janezic and Rupnik [11], i.e. 2% agarose gel for general use and electrophoresis run for 2.5 to 3 hours at 100 V. This PCR ribotyping screening method enables detection of clonal strains but does not precisely identify the PCR ribotype.

One isolate per PCR ribotype per sample was further analysed by capillary-based PCR ribotyping [12], using oligonucleotide primers originally described by Stubbs et al. [13]. Toxinotyping and binary toxin testing was performed as described previously [14,15].

A comparison was made between the PCR ribotypes detected in the current study with those predominantly found in other sources. Sources and settings included the European healthcare system [2,16], animal reservoirs within Europe [8], European-based reports on food other than potato but excluding the seafood [8] and potato sampled in Slovenia [6].

### Whole genome sequencing and sequence analysis

Strains of ribotypes that were isolated at least twice were further investigated by whole genome sequencing (WGS) and analysis using the BioNumerics software v7.5 (Applied Maths, Belgium). Briefly, genomic DNA was quantitated using a Quant-iT dsDNA High-Sensitivity Assay Kit (Thermo Fisher) and a SYNERGY H1 microplate reader (Agilent). Sequencing libraries were prepared from 1 ng of DNA using the Nextera XT DNA Library Prep kit (Illumina), and library concentrations were normalised using AMPure XP beads as described by the manufacturer (Beckman Coulter). Sequencing was performed on the Illumina MiSeq platform with MiSeq v3 600-cycles kit or on the Illumina NextSeq 500 platform with NextSeq 550 High-Output v 2.5 300-cycles kit.

To investigate relationships between *C. difficile* isolates, a whole genome single nucleotide variant analysis (wgSNV) was performed with raw reads mapped to the reference strain of *C. difficile* CD 630 (GenBank accession AM180355.1). The SNV calling was performed using the strict SNV filtering, removing all SNVs with at least one unreliable base, i.e. N, an ambiguous base or gap and non-informative SNVs. SNVs were called if they had at least 5x coverage, once in both directions, and a minimum distance of 12 bp between SNVs.

MEGA10 software was used to construct dendrograms based on retained SNVs, and matrices indicating pairwise SNVs [17].

### Statistical analysis

The proportions of positive samples were analysed by R version 3.4.4 (R Foundation, Vienna, Austria) using Fisher’s exact test. Statistical significance was defined as p < 0.01.

## Results

### Positivity rate of *Clostridioides difficile* in potato samples and production origin of contaminated potatoes

In total, 153 samples of potatoes or potato swabs were collected across 12 European countries and 147 were included in the final analysis (Table 1; Supplementary Table S1); six samples were excluded because of presumptive laboratory cross-contamination. The number of collected samples per country ranged from six (Ireland) to 29 (UK) and were collected from five to 20 retailers per country. Sampling was done between January and July 2018, but most samples were collected in June and July (121/147; 82.3%). All samples were acquired from retail potatoes; the majority were purchased at supermarkets (104/147; 70.7% samples received from all 12 countries), open food markets (28/147; 19.0% from nine countries) or greengrocers (12/147; 8.2% samples from three countries). One potato came directly from a farm, and one from a roadside food seller; for one sample, no location data were provided. In general, one or two samples (mean: 1.4 samples) were collected at each location, i.e. from a single shop.

**Table 1:** Positivity rate, distribution of PCR ribotypes and toxinotypes of *Clostridioides difficile* in potato samples, 12 European countries, 2018

European Region^a^	Country	Number of sampled locations (n)	Number of collected samples (n)	Number of positive samples (n)	Proportion of positive samples (%)	95% CI	PCR ribotypes	Toxinotype
North	Ireland	5	6	3	50.0	11.8–88.2	005	0
							014	0
							078	V (BTb+)
							081	0
							127	VI (BTb+)
							Unknown 1	Tox−
	Sweden	7	9	1	11.1	0.3–48.3	023	IV (BTb+)
							029	0
							276	0
							625	0
	United Kingdom	20	29	1	3.4	0–17.8	010	Tox−
South	Greece	10	14	3	21.4	4.7–50.8	014	0
							917	Tox−
							918	Tox−
	Italy	13	17	3	17.6	3.8–43.4	001	0
							023	IV (BTb+)
							056	XII
							078	V (BTb+)
							912	Tox−
							916	Tox−
							919	Tox−
	Spain	6	10	6	60.0	26.2–87.8	020	0
							126	V (BTb+)
							131	0/v (BTb+)
							204	Tox−
							255	XII
West	Austria	10	12	1	8.3	0.2–38.5	020	0
							027	III (BTb+)
							106	0
							126	V (BTb+)
							276	0
	France	11	15	2	13.3	1.7–40.5	010	Tox–
							015	0
							029	0
							126	V (BTb+)
							128	Tox−
	Netherlands	7	9	1	11.1	0.3–48.3	014	0
East	Poland	9	10	5	50.0	18.7–81.3	002	0
							003	0
							005	0
							018	0
							023	IV (BTb+)
							027	III (BTb+)
							913	Tox−
							914	Tox−
	Romania	5	7	7	100.0	59.0–100	002	0
							024	0
							126	V (BTb+)
							174	0
							207	0
							864	XII
							914	Tox−
							915	Tox−
							Unknown 2	Tox−
	Slovakia	7	9	0	NA	0–33.6	NA	NA
**Total**	**12**	**110**	**147**	**33**	**22.4**	**16.0–30.1**	**36 and 2 unknown**	**7**

Btb: presence of *Clostridium difficile* binary toxin; CI: confidence interval; Tox−: nontoxigenic strain.

^a^ Participating countries equally represent the four European subregions as defined by [10].

Overall, 33 of 147 collected samples (22.4%; 95% CI: 16.0–30.1) tested positive for *C. difficile*. All samples from Slovakia were negative (n = 9) and all samples from Romania were positive (n = 7). The positivity rate in other countries varied from 3.4% (1/29; 95% CI: 0–17.8) in the UK to 60.0% in Spain (6/10; 95% CI: 26.2–87.8) (Table 1).

For 134 of 147 (91.2%) samples, the information on country of production origin (domestic product or imported product) was available. Of these, only 13 (9.7%) potato samples were imported. There was no significant difference between the positivity rate of *C. difficile* in imported vs non-imported (p = 0.47) potatoes. Most imported potatoes originated from other European countries (n = 10) and three originated from other continents (United States, Asia (Israel) and Africa (Egypt)) (additional information on the production origin of *C. difficile*-positive samples is presented in Supplementary Table S1).

Of all collected samples, more than half were visibly covered with soil remnants, moderately for 25.9% (38/147) and excessively for 31.3% (46/147). There was a significant difference in the proportion of *C. difficile*-positive samples between visibly clean potatoes (3.2%; 2/63) vs those moderately (36.8%; 14/38) or excessively (37.0%; 17/46) (p < 0.01) covered with soil.

### PCR ribotyping and toxinotyping of potato isolates

From a single *C. difficile*-positive sample, up to 12 colonies were further cultivated, resulting in a total of 504 isolates screened with crude PCR ribotyping to identify identical PCR ribotype profiles. One isolate per PCR ribotype profile per positive sample (n = 61) was further used for capillary-based PCR ribotyping. This revealed two additional identical ribotype profiles and, based on the criterion ‘one PCR ribotype per sample’, the number of unique isolates was reduced from 61 to 59. Isolates were distributed into 38 different PCR ribotypes, of which two were not assigned a RT number and are listed as ‘Unknown’ (Table 1, Table 2, Supplementary Table S1). Up to five *C. difficile* PCR ribotypes were found within a single sample (Supplementary Table S1).

**Table 2:** Overview of *Clostridioides difficile* PCR ribotypes detected on potatoes in the COMBACTE-CDI potato study, 2018, compared with published works on *C. difficile* PCR ribotypes across diverse reservoirs in Europe

PCR ribotypes	Global prevalence of ribotypes in various studies	COMBACTE-CDI potato study^a^	Published works on *Clostridioides difficile*				
			EUCLID ribotype distribution^b^ [16]	ECDC [2]	European countries [8]	European countries [8]	Slovenia [6]
Sampling period		2018	Dec 2012–Aug 2013	2016	Longer time intervals	Longer time intervals	2016–17
Sample source		Potato	Humans	Humans	Animals	Food other than potato (excluding seafood)	Potato
001	Common	1	Detected	Detected	NF/T	Detected	Detected
002		2	Detected	Detected	Detected	NF/T	NF/T
003		1	NF/T	NF/T	Detected	Detected	
005		2	Detected		Detected	NF/T	
010		3	Detected		Detected	NF/T	
014		3	Detected	Detected	Detected	Detected	Detected
015		1	Detected	NF/T	Detected	Detected	NF/T
018		1	Detected	NF/T	NF/T	NF/T	NF/T
020		2	Detected	Detected	Detected		Detected
023		4	Detected	NF/T	Detected		Detected
027		2	Detected	Detected	NF/T		Detected
078		2	Detected	Detected	Detected	Detected	NF/T
126		7	Detected	NF/T	Detected	NF/T	Detected
106		1	NF/T		NF/T		NF/T
056		1					
081	Less common to rare	1					
024		1					
029		2					
127		1					
128		1					
131		1					
174		1					
204		1					
207		1					
255		1					
276		2					
625		1					
864		1					
912–919^c^	Rare, divergent	9					
Unknown 1 and 2	Unknown	2					
Other common ribotypes	Common	NA	026, 140, 009, 070, 356, 017, 011, 012		045, 066, 033	012, 045, 053	150

NA: not applicable; NF/T: not found or tested.

^a^ All 504 COMBACTE-CDI potato isolates were first screened with crude PCR ribotyping, which enables detection of identical band profiles but not the assignment of PCR ribotype. Based on criterium of one ribotype per sample, the number of isolates was reduced to 59 strains shown in this table.

^b^ This source reports the 10 most common *C. difficile* ribotypes combined for four geographic regions.

^c^ Of these ribotypes, only RT 914 included two isolates, others were represented by a single isolate. In RT 912, seven strains from various countries were isolated but because of potential but not proven laboratory contamination, only the first isolate was included in the final analysis

**Figure 1:**
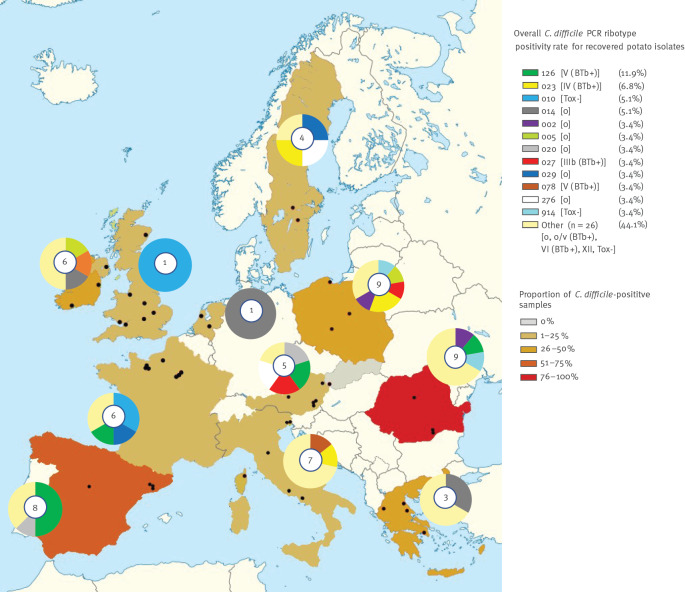
Positivity rate in retail potatoes and the geographical distribution of PCR ribotypes and toxinotypes of *Clostridioides difficile*, 12 European countries, 2018

The most common PCR ribotypes were 126, 023, 010 and 014 (Figure 1 and Table 2). Some PCR ribotypes (from RT 912 to RT 919) were new to the Leeds *C. difficile* database (maintained by M Wilcox laboratory, University of Leeds, Leeds, UK), which contained over 900 different PCR ribotypes at the time of this study (September 2018); two additional PCR ribotypes could not be determined by capillary-based PCR ribotyping (Unknown 1 and 2). The diversity of PCR ribotypes was substantial and overlap between samples or countries was moderate. Only 12 of 38 PCR ribotypes appeared more than once: four PCR ribotypes more than twice (RTs 126, 023, 010, 014) and eight PCR ribotypes twice (RTs 002, 005, 020, 027, 029, 078, 276, 914). The common PCR ribotypes were found in more than one European region (Figure 1). No pattern of ribotype distribution was apparent across the four geographical regions of Europe.

Btb: presence of *Clostridium difficile* binary toxin; CI: confidence interval; Tox−: non-toxinogenic strain

Black dots show potato sampling sites. Pie charts show the proportion of the most common PCR ribotypes in individual countries. The number in the centre of pie chart is the number of isolates included in data analysis. Toxinotypes are listed in square brackets. The legend also shows percentage (%) of common PCR ribotypes among all 59 isolates from all countries. Other PCR ribotypes (n = 24) were detected only in a single sample: 001, 003, 015, 018, 024, 056, 081, 106, 127, 128, 131, 174, 204, 207, 255, 625, 864, 912, 913, 915, 916, 917, 918, 919. Two non-toxigenic PCR ribotypes could not be determined by the capillary-based PCR ribotyping; they are placed in the group Other.

Seven toxinotypes, some of them harbouring also the marker gene (BTb) for binary toxin, were identified, including 0, 0/v (BTb+), III (BTb+), IV (BTb+), V (BTb+), VI (BTb+) and XII and nontoxigenic isolates. Up to four different toxinotypes could be identified within a single sample. Most of the detected PCR ribotypes belonged to toxinotype 0 (n = 16), followed by non-toxigenic PCR ribotypes (n = 13). All new and undetermined PCR ribotypes were non-toxigenic.

### Whole genome sequence analysis and absence of clonal spread

**Figure 2:**
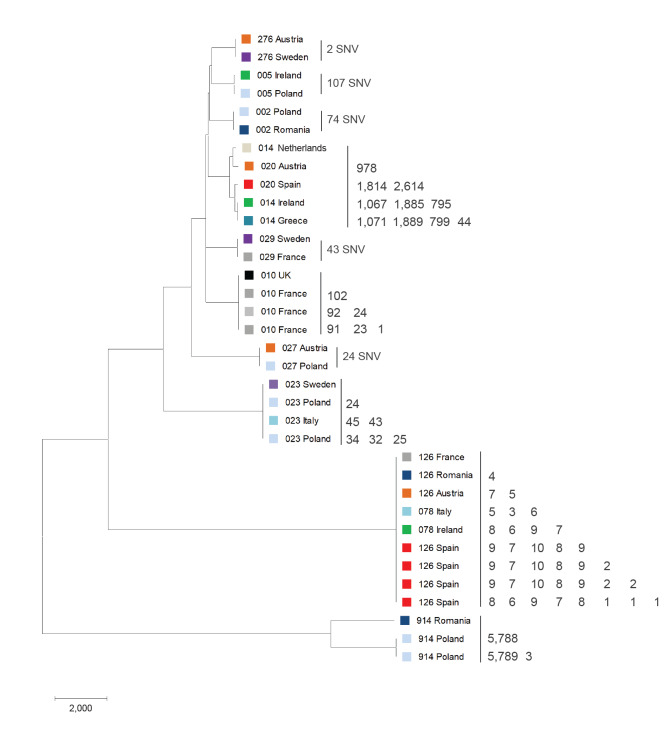
Comparison of whole genome sequences for PCR ribotypes represented by two or more *Clostridioides difficile* strains, 12 European countries, 2018

Twelve of 38 PCR ribotypes included two or more strains (n = 35 strains), which were further analysed by WGS (Figure 2). Strains clustered according to the ribotype and as expected, RT 014 and RT 020 were found in the same cluster, and similarly so for RT 078 and RT 126. Clustering of RT 914 strains is probably because of a recent divergence of *C. difficile* clades, as deduced from the position in the genome similarity tree.

SNV: single nucleotide variation.

Ribotypes are designated by numbers. Countries are shown also by colour code. The matrices on the right side show the SNV differences between genomes within the same PCR ribotype (calculated for each PCR ribotype individually).

Mostly, strains within a given ribotype showed a substantial number of SNVs (Figure 2), but several examples of genetically related strains were also detected. Most of the strain pairs were from samples from the same country (Spain/RT 126 and France/RT 010). Additionally, we detected two genetically linked strains of RT 276 with only 2 SNV differences that were not isolated in the same country (from Austria and Sweden).

## Discussion

We report here the positivity rate of *C. difficile* contamination of potatoes sampled across 12 European countries. The high potato contamination rates (≥ 10% of all samples in 9/12 countries) could have potential public health relevance. Potatoes are typically washed, peeled and cooked before eating, which reduces the risk that they could be a direct source of infection. Nevertheless, potatoes can serve as a vector for introducing *C. difficile* spores in the household environment and/or food chain, where they could persist. After such indirect exposure, it is possible that ingested *C. difficile* could multiply, particularly in the presence of disrupted or unmature microbiota of a sensitive host (human or pet). In this way, potatoes could have a role in the transmission of *C. difficile* between community reservoirs.

The overall proportion of *C. difficile*-positive potatoes (22.4%) is similar to that reported in Slovenia (25.7%; 18/70) in 2015–17 [6], but lower than that measured in Australia (53.3%) [5] in 2015. However, different contamination rates were found in our study across the 12 countries, ranging from 0.0% to 100% (95% CI: 59.0–100) (Table 1). There was no correlation between contamination rate and the number of samples per country, and no geographical pattern of high/low contamination rate was observed (Figure 1). One to 5 days passed between sampling and testing in the laboratory. No association between transit time and testing result (positivity) was observed.

The PCR ribotype diversity was high and 38 PCR ribotypes were found in a total of 33 positive potato samples. These PCR ribotypes were categorised into three groups (Table 2) based on global prevalence. As shown in the Table 2 and references cited therein, some ribotypes are commonly found in humans, animals and the environment. Another group of PCR ribotypes is less commonly present and hence not well documented in the published literature. The third group represents divergent clusters, similar to those typically isolated from soil and currently rarely reported [18].

Twelve PCR ribotypes found on potatoes were among most common clinically relevant PCR ribotypes in the large European study performed from December 2012 to August 2013 (EUropean, multi-centre, prospective bi-annual point prevalence study of *CLostridium difficile* Infection in hospitalised patients with Diarrhoea (EUCLID)) [16]; six of them were also among the most common PCR ribotypes in European hospitals in 2016 [2] (Table 2). The overlap with the animal reservoir is also considerable; 10 PCR ribotypes often found in different animals were present on potato across Europe. The PCR ribotype with highest prevalence was 078/126. This ribotype is prevalent in farm animals, mainly pigs, on a global scale. Thus, recovery of *C. difficile* from potatoes probably reflects the use of animal-based soil manure. Five PCR ribotypes were shared with those found previously on food [8] and six were shared with PCR ribotypes found previously on potatoes in Slovenia [6]. These results again confirm that some PCR ribotypes are more likely to spread across several hosts and reservoirs than others.

Confounding is a clear risk even with a relatively large sample size as used here. The sources of contamination are only speculative and very likely multiple. For instance, a single sample with five different ribotypes could be suggestive of hand transmission in the retail store and/or during the transport. Another likely source of different ribotypes is the soil. Many clinically common ribotypes (RTs 014, 010, 023) are also commonly found in soil samples [19]. Divergent *C. difficile* lineages are also typically soil-associated [18]; RT 912 to RT 919 are examples of divergent representatives found on potatoes from Romania, Poland, Greece and Italy. In this study, a soil-contaminated potato sample was significantly more likely to be contaminated with *C. difficile*.

Genomic comparison of our strains with available genomes was not the aim of this study and such comparison would be very time consuming if performed properly on hundreds of published genomes. We used WGS in current study to show if and to what extend were the strains from the same ribotype related to each other. Within a given PCR ribotype, strains usually differed in a large number of SNVs (Figure 2), but some genetic relatedness was detected in RT 276, RT 010 and RT 126. A difference in one or two SNVs is commonly accepted as genetic relatedness in *C. difficile* and is most likely a result of direct transmission or a common source [20]. Only a single related strain pair included isolates from two different countries, Austria and Sweden (RT 276). The Austrian potato sample was bought and analysed in February 2018 and the Swedish sample purchased several months later in June. Both RT 276 strains were isolated from samples contaminated with multiple ribotypes (Table 1; Austria and Sweden) which, together with a four-month gap between the laboratory analysis of both RT 276 positive samples, makes contamination highly unlikely. Such findings of genetically related strains in the large geographical or temporal distances have been previously described [19,21]. It is unlikely that both samples were contaminated from common source, as both potatoes were domestic and not imported. The result suggests clonal spread of a strain of RT 276 between these two countries (and potentially elsewhere) by unknown transmission routes. RT 276 is not commonly found in Europe. Excluding COMBACTE CDI strains, only six and 38 RT 276 strains are present in the Leeds and Slovenian ribotype collections (M Rupnik laboratory), respectively. In both collections, RT 276 strains started to be recognised in the same year (2010). Strains were predominantly isolated from humans (n = 42) in more than 3 countries (UK and 3 non-UK studies in the Leeds collection; Slovenia, and North Macedonia in the Rupnik laboratory collection), but also from potato (n = 1 in Albania) and canine (n = 1 in Slovenia) sources.

A threshold of 10 or more SNVs is commonly accepted for genetically unrelated *C. difficile*. In the group of RT 078/126, genetic distance was lower than this and we concluded a possible spread of genetically similar but nonclonal strains. Similar clusters have been described already for RT 078 transmissions between humans and animals in a large worldwide collection [22].

During the study, seven isolates designated as PCR ribotype 912 were obtained from five different countries, and all were clonal by WGS analysis. It is difficult to confidently interpret this as either representing clonal spread or laboratory contamination. While we cannot absolutely exclude the possibility of laboratory contamination, there was a three-month time interval between the processing of the first and second RT912 positive sample, with a large number of RT912 negative samples in between. However, in the final analysis presented here, the RT912 is included only with a single isolate that was obtained from a first RT912 positive sample (Italy, April 2018). All other six samples with this ribotype were excluded from the analysis and are summarised in Supplementary Table S2, which shows an overview of all RT 912 strains detected in our study.

## Conclusion

In summary, potato is a food commonly contaminated with *C. difficile*, although the positivity rates across countries varied substantially. The absence of clonal clusters indicates that there are no clearly successful clonal strains, although our sample size was modest. For the large cluster of RT 912, we could not rule out the possible laboratory contamination. However, the prevalent PCR ribotypes we detected (014/020, 078/126, 010) overlap with the *C. difficile* population that is found in humans, animals and soil. The role of food in *C. difficile* transmission chains still needs to be clarified. We believe that very large sample sizes will be needed, ideally with repeat sampling of specific places to detect sporadic contamination, and to understand fully the extent and relevance of *C. difficile* in foods. Potatoes could serve as a carrier of spore spread between countries and in the contamination of domestic environments. Such constant exposures combined with temporarily disturbed gut microbiota (impaired colonisation resistance) may then contribute to the onset of community associated CDI.

## Supplementary Data

181000 Supplement
