# Supplementary material for: Clostridioides difficile positivity rate and PCR ribotype distribution on retail potatoes in 12 European countries, January to June 2018
Source: Euro Surveill. 2022 Apr 14;27(15):2100417. doi: 10.2807/1560-7917.ES.2022.27.15.2100417 (PMC9012089; doi:10.2807/1560-7917.ES.2022.27.15.2100417)
Supplement: Supplement [file 21-00417_RUPNIK_Supplement.pdf]

Clostridioides difficile prevalence and PCR ribotype distribution on retail potatoes in 12 European countries, 2018

Disclaimer

"This supplementary material is hosted by *Eurosurveillance* as supporting information alongside the article 'Clostridioides difficile prevalence and PCR ribotype distribution on retail potatoes in 12 European countries, 2018', on behalf of the authors, who remain responsible for the accuracy and appropriateness of the content. The same standards for ethics, copyright, attributions and permissions as for the article apply. Supplements are not edited by *Eurosurveillance* and the journal is not responsible for the maintenance of any links or email addresses provided therein."

Supplementary Table S1. Information on sampling and *Clostridioides difficile* positive samples in recruited European countries, year 2018.

Country	Number of sampled retail places (n)	Type of retailer	Number of collected samples (n)	Number of cities (n)	Time frame of sampling	Number of sampling occasions (n)	Number of *C. difficile* positive samples	Proportion of *C. difficile* positive samples	95% CI (%)	PCR ribotype	Toxinotype	Sample designation	Origin of collected samples[a]
Austria	10	Grocery store<br>Food market	11<br>1	4	12.1.-<br>4.6.2018	7	1	8.3	0.2-38.5	020<br>027<br>106<br>126<br>276	0<br>III (BTb+)<br>0<br>V (BTb +)<br>0	CP012	Austria
France	11	Grocery store<br>Food market	8<br>7	7	18.6.-<br>16.7.2018	9	2	13.3	1.7-40.5	010<br>015<br>029<br>126<br>128	Tox –<br>0<br>0<br>V (BTb +)<br>Tox -	CP033,<br>CP034<br>CP034<br>CP034<br>CP034<br>CP034	France
Greece	10	Grocery store<br>Food market	10<br>4	4	27.6-<br>5.7.2018	7	3	21.4	4.7-50.8	014<br>917<br>918	0<br>Tox -<br>Tox -	CP128<br>CP140<br>CP129	Greece[2]<br>Cyprus[1]<br>France
Ireland	5	Grocery store<br>Food market<br>Farm	3<br>2<br>1	2	14.7.-<br>21.7.2018	4	3	50.0	11.8-88.2	005<br>014<br>078<br>081<br>127<br>unknown	0<br>0<br>V (BTb +)<br>0<br>VI (BTb +)<br>Tox -	CP161<br>CP162-168<br>CP162-168<br>CP158<br>CP161<br>CP162-168	Ireland
Italy	13	Grocery store<br>Food market	14<br>2	5	14.4-<br>3.7.2018	7*	3	17.6	3.8-43.4	001<br>023	0<br>IV (BTb +)	CP018<br>CP066	Italy

		Greengrocer	1							056<br>078<br>912<br>916<br>919	XII<br>V (BTb +)<br>Tox –<br>Tox –<br>Tox -	CP018<br>CP018<br>CP018<br>CP028<br>CP028	
Netherlands	7	Grocery store<br>Greengrocer<br>No data	5<br>3<br>1	2	15.6.-<br>12.7.2018	4	1	11.1	0.3-48.3	014	0	CP119	Netherlands[1]<br>France<br>Egypt<br>Israel
Poland	9	Grocery store<br>Food market	7<br>3	3	9.6.,<br>27.6.2018	2	5	50.0	18.7-81.3	002<br>003<br>005<br>018<br>023<br>027<br>913<br>914	0<br>0<br>0<br>0<br>IV (BTb +)<br>III (BTb +)<br>Tox –<br>Tox -	CP136<br>CP134<br>CP132<br>CP135<br>CP134, 136<br>CP132<br>CP045<br>CP045	Poland[4]<br>France<br>Spain<br>Greece[1]
Romania	5	Grocery store<br>Food market<br>Food stand by the road	2<br>4<br>1	3	15.6.-<br>12.7.2018	5	7	100.0	59.0-100	002<br>024<br>126<br>174<br>207<br>864<br>914<br>915<br>unknown	0<br>0<br>V (BTb +)<br>0<br>0<br>XII<br>Tox –<br>Tox –<br>Tox -	CP058<br>CP061<br>CP059<br>CP057<br>CP059<br>CP059<br>CP170<br>CP060<br>CP169	Romania[6]<br>USA[1]
Slovakia	7	Grocery store<br>Food market	6<br>3	1	11.1,<br>11.7.2018	2	0	NA	0-33.6	NA	NA	NA	Slovakia<br>France
Spain	6	Grocery store<br>Greengrocer	2<br>8	4	23.6.-<br>28.7.2018	6	6	60.0	26.2-87.8	020<br>126<br><br>131<br>204<br>255	0<br>V (BTb +)<br><br>0/v (BTb +)<br>Tox –<br>XII	CP154<br>CP148, 149, 150, 151<br>CP149<br>CP148<br>CP147	No information[6]<br>Spain
Sweden	7	Grocery store	9	2	19.6.,<br>27.6.2018	2	1	11.1	0.3-48.3	023<br>029<br>276<br>625	IV (BTb +)<br>0<br>0<br>0	CP087<br>CP087<br>CP087<br>CP087	Sweden
UK	20	Grocery store<br>Food market	27<br>2	8	12.1.-<br>30.7.2018	16	1	3.5	0-17.8	010	Tox –	CP053	UK[1]<br>Egypt<br>No information
Total	110	NA	147	45	11.1.-<br>30.7.2018	71	33	22.5	16.0-30.1	38	7 toxinotypes	NA	NA

[a]-Number of *C. difficile* positive samples from specified country of origin.

NA-not applicable.

*-minimal number of sampling occasions- information on exact sampling date not available for all samples.

Supplementary Table S2. Overview of all RT 912 strains detected in our study. Sample CP018 was the only one included in the final analysis, while other six were excluded due to potential but not confirmed laboratory contamination.

sample designation	sampling date	received in the lab	date of experiment	campling (country)	campling (City)	store	country of origin (original)	imported/domestic	received sample type	soil contamination (1-visible; 2- some, 3-none)	PCR ribotype Leeds (CE)	toxinotype	BTb	ZZV designation
CP096	27.06.2018	2.07.2018	2.07.2018	Sweden	Linköping	City Gross	Sweden	domestic	swab	3	912	tox-	BTB-	ZZV18-9342
CP106	9.07.2018	13.07.2018	14.07.2018	UK (England)	Cambridge	CO-OP	no data	no data	swab	3	912	tox-	BTb-	ZZV18-9387
CP109	3.07.2018	6.07.2018	6.07.2018	Italy	Napoli	Minimarket -pollena trocch	Italy	domestic	swab	2	912	tox-	BTb-	ZZV18-9364
CP110	3.07.2018	6.07.2018	6.07.2018	Italy	Napoli	Sisa-casoria	Italy	domestic	swab	2	912	tox-	BTb-	ZZV18-9363
CP117	11.07.2018	16.07.2018	17.07.2018	Netherlands	Wageningen	Albert Heijn	Egypt	imported	swab	3	912	tox-	BTb-	ZZV18-9522
CP127	2.07.2018	10.07.2018	10.07.2018	Greece	Athens	Kolitsidas	Greece	domestic	swab	2	912	tox-	BTb-	ZZV18-9381
CP018	14.04.2018	16.04.2018	17.04.2018	Italija	Fernetti	Confort il discount Aliment	Italy	domestic	potato	1	912	tox-	BTb-	ZZV18-9317
